# Supplementary material for: Spatial domain detection using contrastive self-supervised learning for spatial multi-omics technologies
Source: bioRxiv. 2024 Feb 4:2024.02.02.578662. Preprint. [Version 1] doi: 10.1101/2024.02.02.578662 (PMC10862910; doi:10.1101/2024.02.02.578662)
Supplement: Supplement 1 [file NIHPP2024.02.02.578662v1-suppiement-1.pdf]

bioRxiv preprint doi: https://doi.org/10.1101/2024.02.02.578662; this version posted February 4, 2024. The copyright holder for this preprint (which was not certified by peer review) is the author/funder, who has granted bioRxiv a license to display the preprint in perpetuity. It is made available under aCC-BY 4.0 International license.

Supplementary Figures

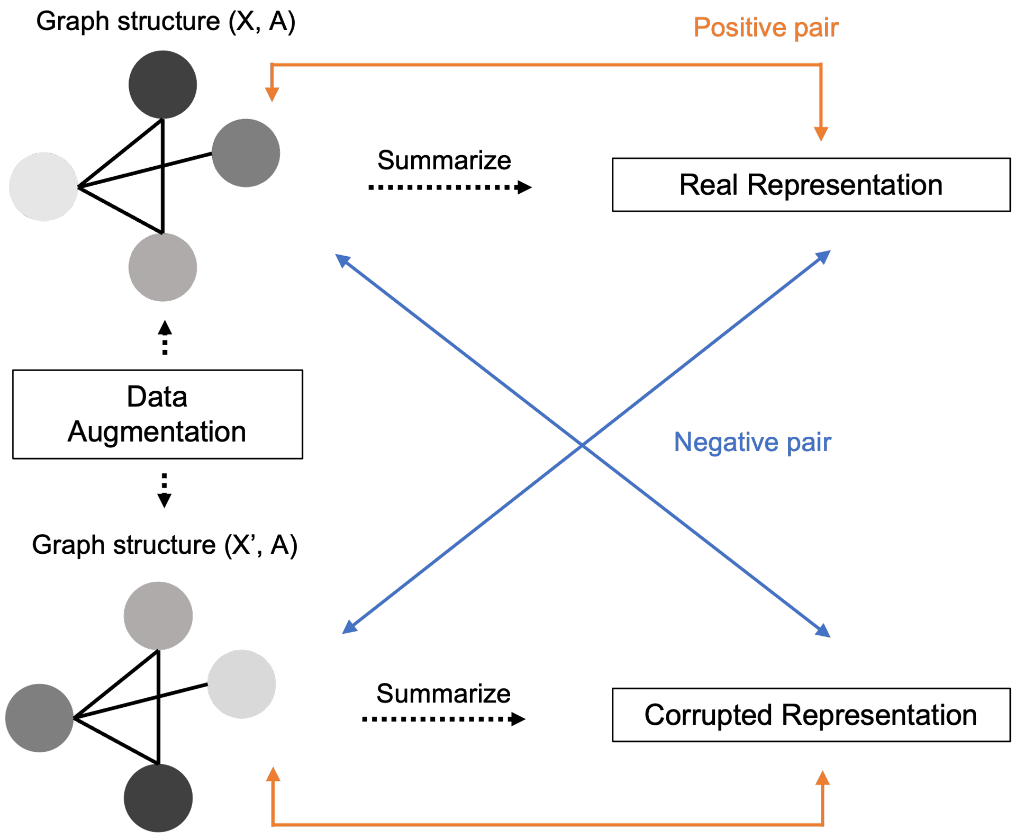

Figure S1: Contrastive self-supervised learning. Contrastive self-supervised learning is illustrated in this figure, demonstrating the refinement of latent representations during training of a graph-based autoencoder model. In the data augmentation step, biological features are randomly shuffled while preserving the distance-based graphs connecting each observation. Real and corrupted local representations are then summarized from these two sets of graph structures using a read-out function. A discriminative score for each pair of spot-patch representations is calculated during each iteration, comparing the spot-level latent embeddings with the summarized local context, respectively.

bioRxiv preprint doi: https://doi.org/10.1101/2024.02.02.578662; this version posted February 4, 2024. The copyright holder for this preprint (which was not certified by peer review) is the author/funder, who has granted bioRxiv a license to display the preprint in perpetuity. It is made available under aCC-BY 4.0 International license.

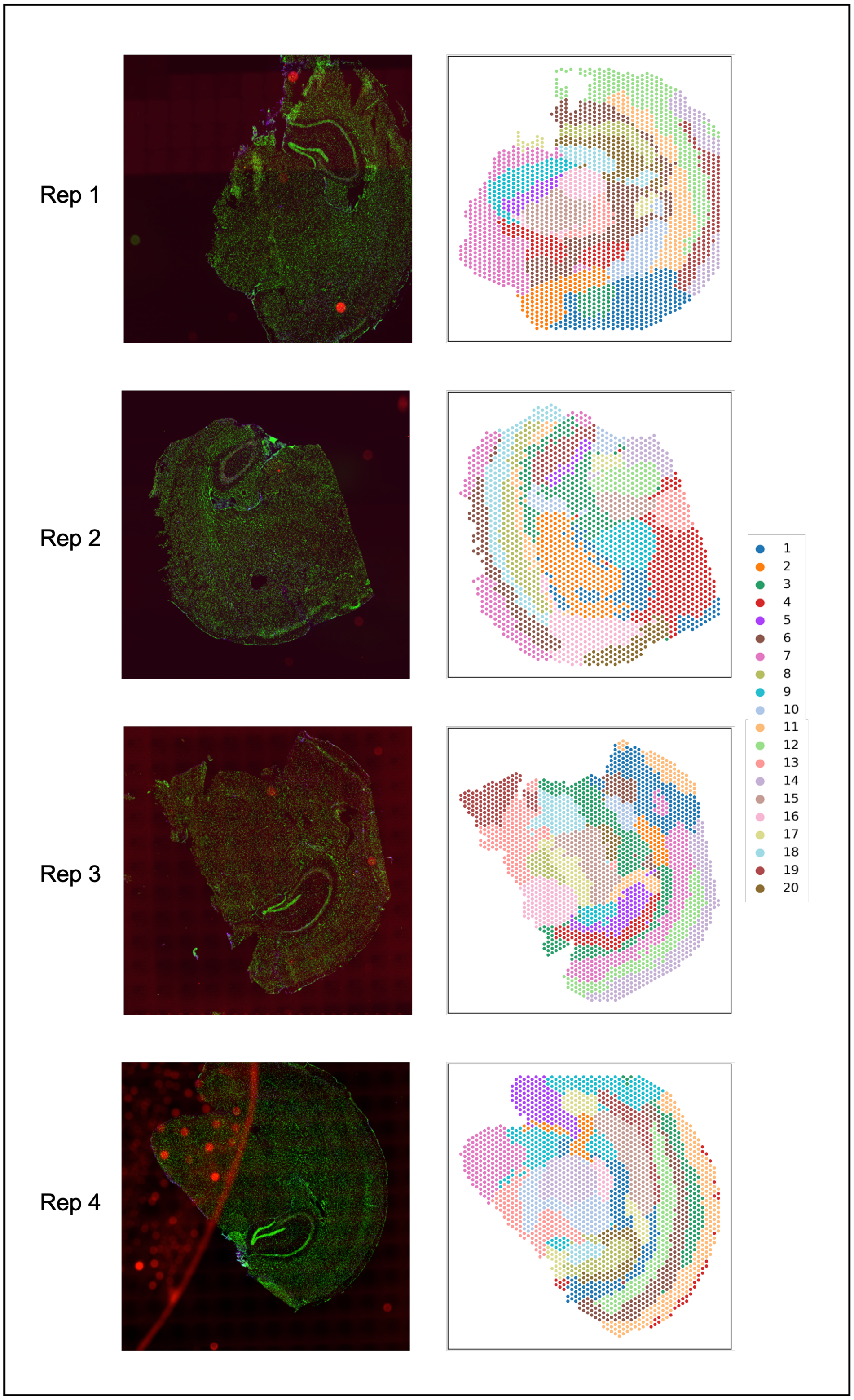

Figure S2: CK-p25 mouse coronal brain tissues measured on the Visium SPG platform across four tissue replicates. For each of the four tissue replicates (rows), the IF staining images of γH2AX protein reproduced from Welch et al. [27] (left column) and the spatial domains detected by Proust for k=20 domains (right column).

bioRxiv preprint doi: https://doi.org/10.1101/2024.02.02.578662; this version posted February 4, 2024. The copyright holder for this preprint (which was not certified by peer review) is the author/funder, who has granted bioRxiv a license to display the preprint in perpetuity. It is made available under aCC-BY 4.0 International license.

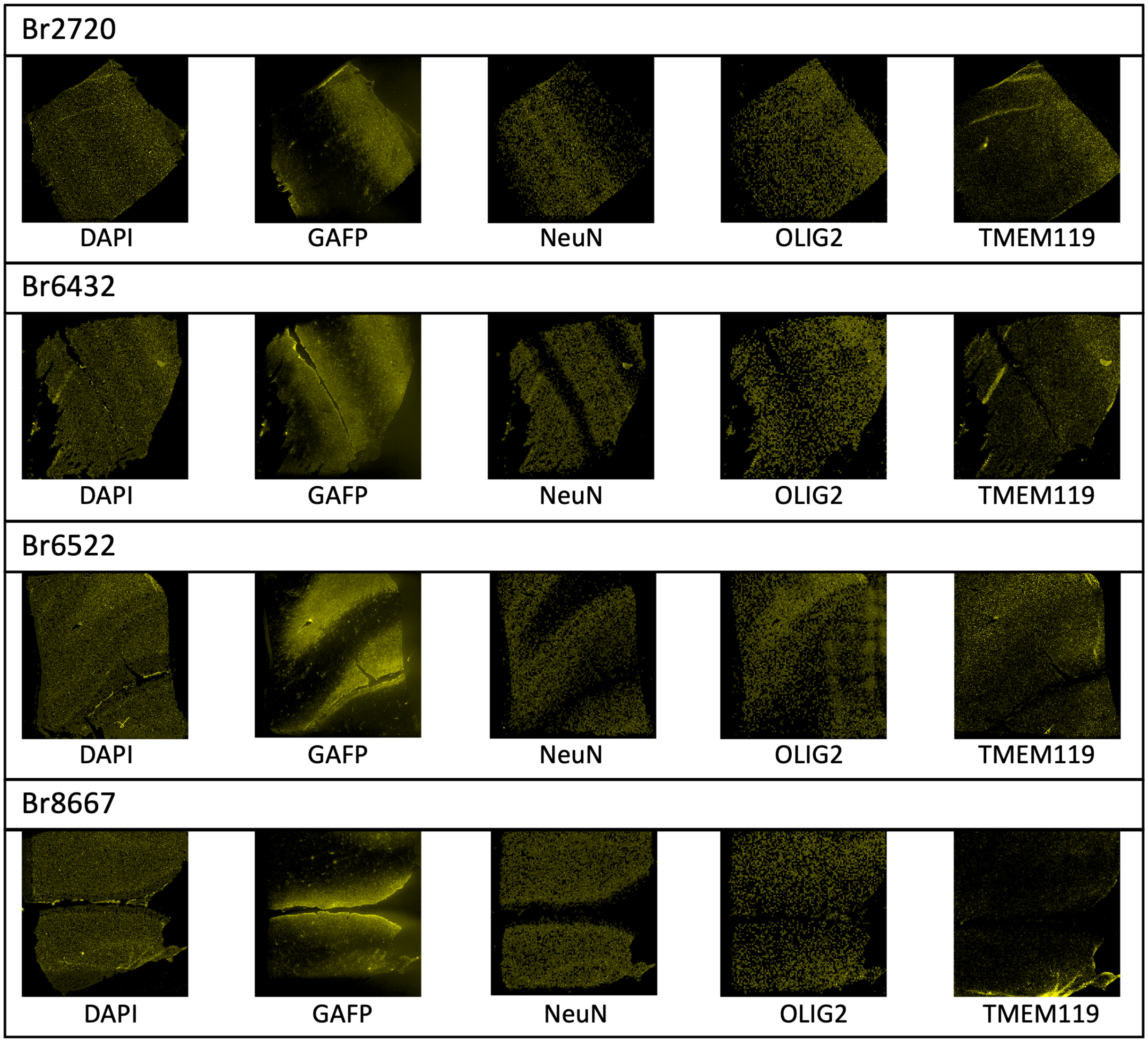

Figure S3: IF images from the Visium SPG human DLPFC samples. IF images of five cell-type channels (DAPI, GAFP, NeuN, OLIG2, and TMEM119) from four Visium SPG human DLPFC samples.

bioRxiv preprint doi: https://doi.org/10.1101/2024.02.02.578662; this version posted February 4, 2024. The copyright holder for this preprint (which was not certified by peer review) is the author/funder, who has granted bioRxiv a license to display the preprint in perpetuity. It is made available under aCC-BY 4.0 International license.

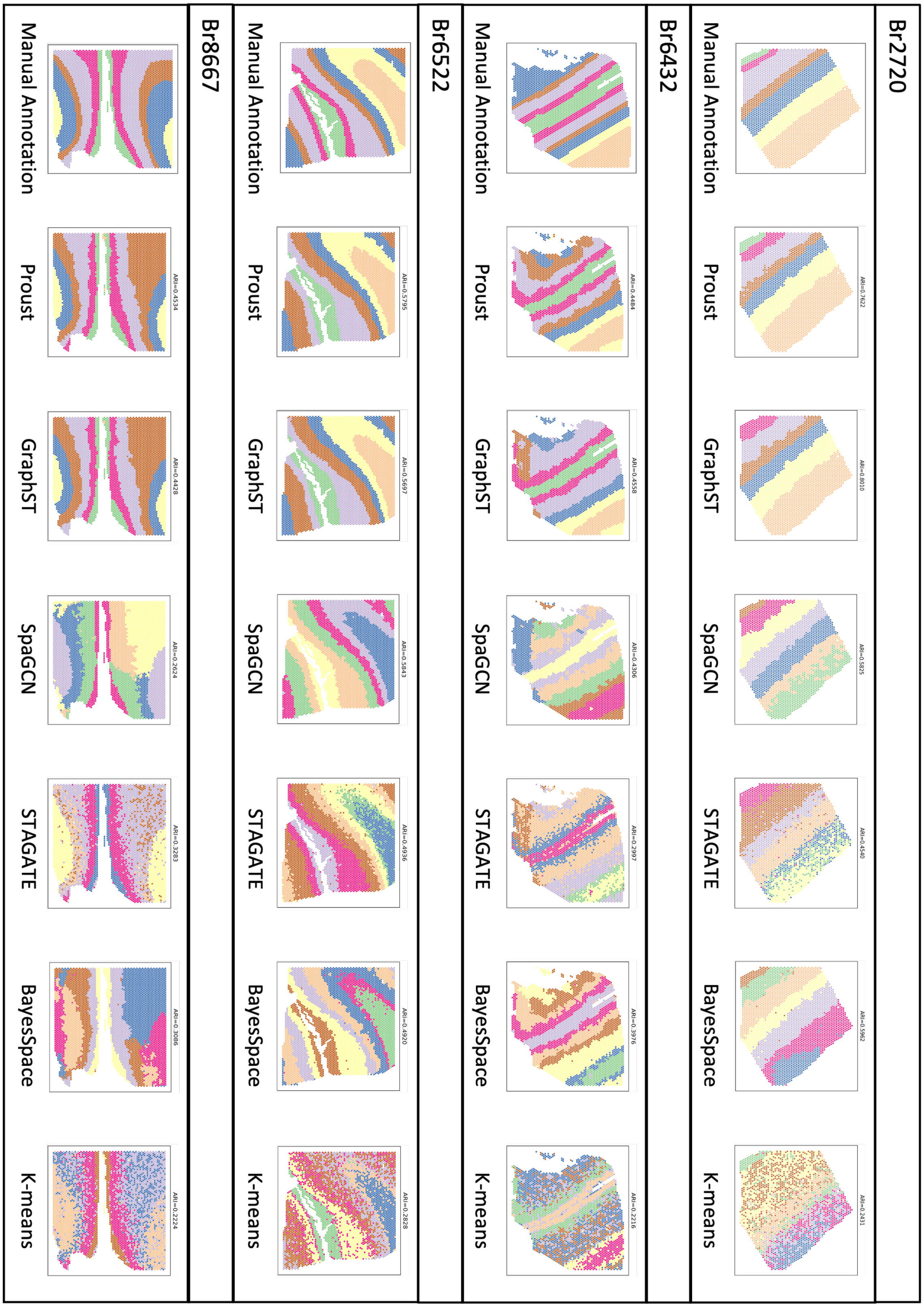

Figure S4: Manual annotations and clustering results from six methods on the Visium SPG human DLPFC samples. Manual annotations and clustering results from six methods on four Visium SPG human DLPFC samples.

bioRxiv preprint doi: https://doi.org/10.1101/2024.02.02.578662; this version posted February 4, 2024. The copyright holder for this preprint (which was not certified by peer review) is the author/funder, who has granted bioRxiv a license to display the preprint in perpetuity. It is made available under aCC-BY 4.0 International license.

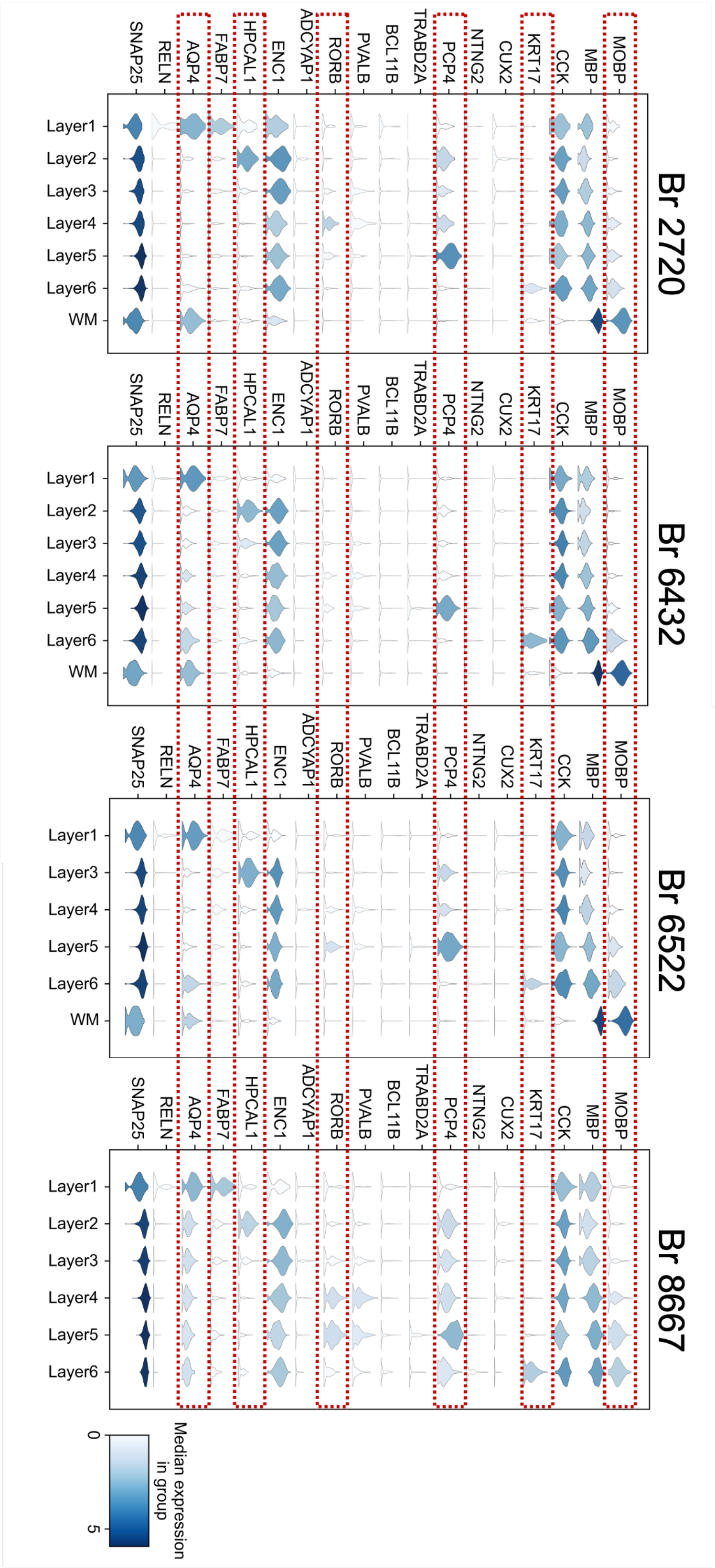

Figure S5: Stacked violin plots of marker genes across annotated clusters generated by Proust for the Visium SPG human DLPFC samples. Stacked violin plots of marker genes across annotated clusters generated by Proust for four Visium SPG human DLPFC samples. Selected marker genes for each layer are boxed.

bioRxiv preprint doi: https://doi.org/10.1101/2024.02.02.578662; this version posted February 4, 2024. The copyright holder for this preprint (which was not certified by peer review) is the author/funder, who has granted bioRxiv a license to display the preprint in perpetuity. It is made available under aCC-BY 4.0 International license.

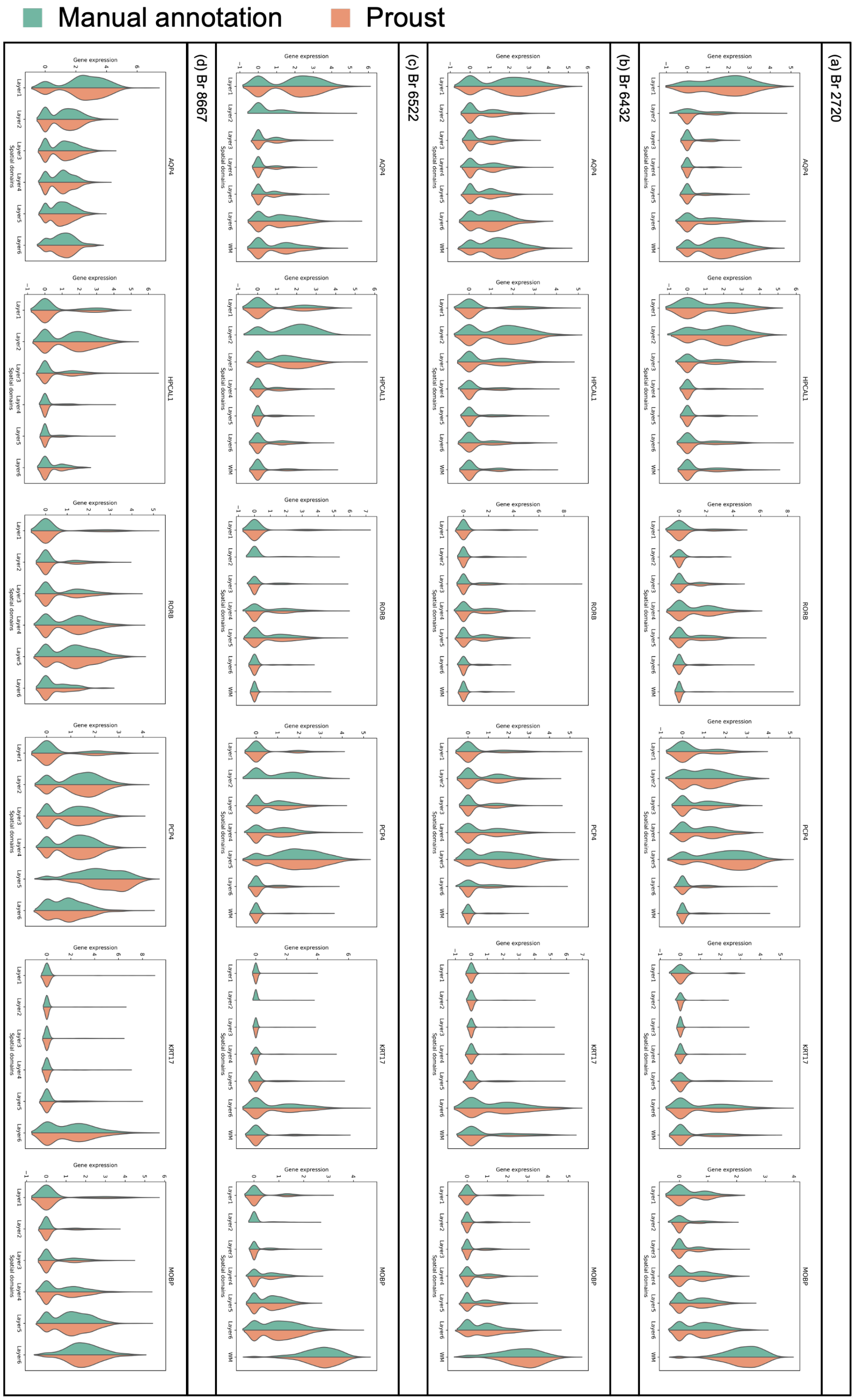

Figure S6: Violin plots of marker genes within clusters identified by Proust and manual annotations of the Visium SPG human DLPFC samples. Violin plots to compare marker gene distributions within clusters identified by Proust and manual annotations of four Visium SPG human DLPFC samples.

bioRxiv preprint doi: https://doi.org/10.1101/2024.02.02.578662; this version posted February 4, 2024. The copyright holder for this preprint (which was not certified by peer review) is the author/funder, who has granted bioRxiv a license to display the preprint in perpetuity. It is made available under aCC-BY 4.0 International license.

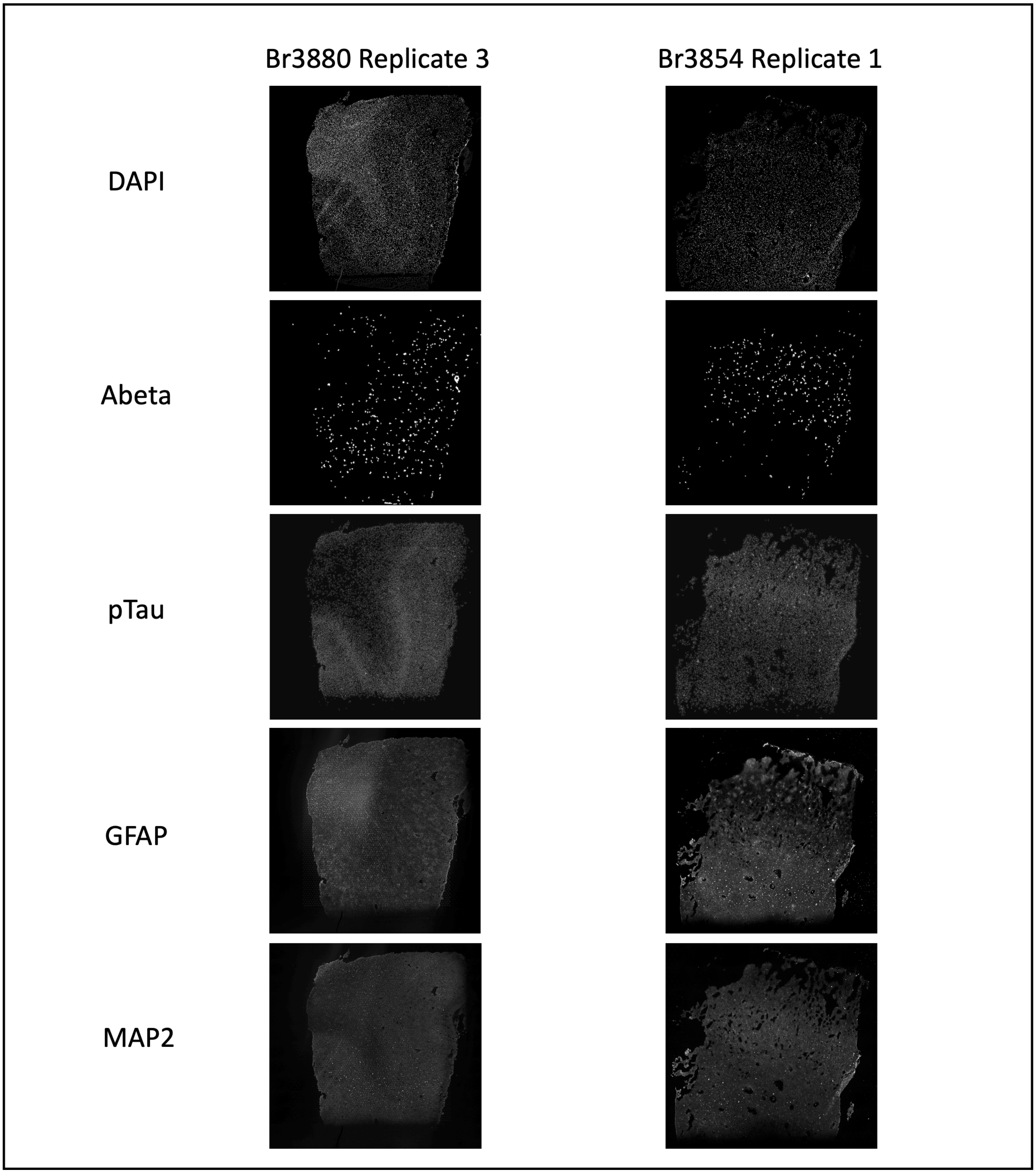

Figure S7: IF images from selected Visium SPG human inferior temporal cortex samples. IF images of five protein channels (DAPI, Abeta, pTau, GFAP, and MAP2) from selected Visium SPG human inferior temporal cortex samples.

bioRxiv preprint doi: https://doi.org/10.1101/2024.02.02.578662; this version posted February 4, 2024. The copyright holder for this preprint (which was not certified by peer review) is the author/funder, who has granted bioRxiv a license to display the preprint in perpetuity. It is made available under aCC-BY 4.0 International license.

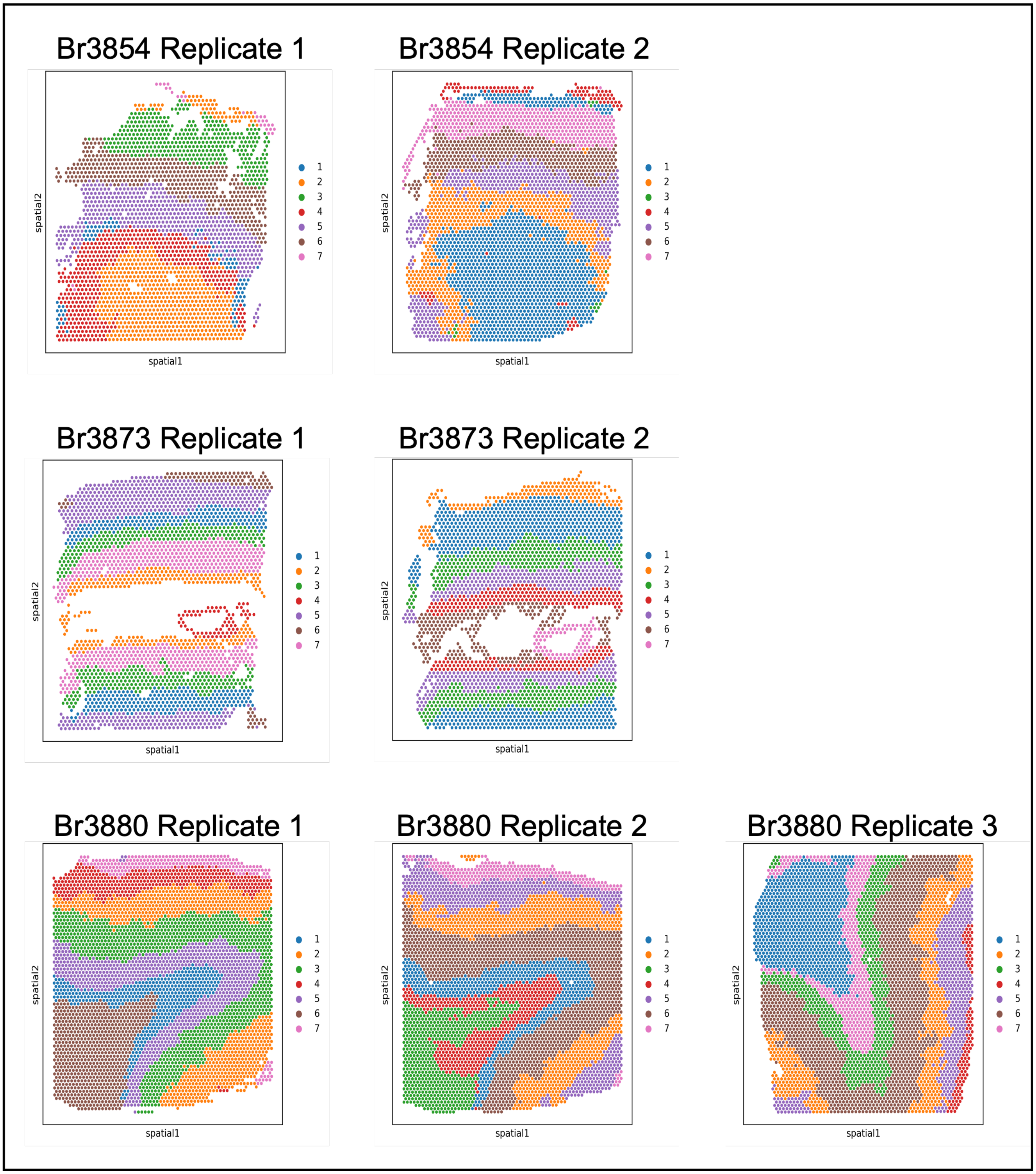

Figure S8: Proust clustering results of the Visium SPG human inferior temporal cortex samples, using five protein channels (DAPI, Abeta, pTau, MAP2, and GFAP), top 30 PCs from reconstructed gene expression, top 5 PCs from reconstructed extracted image features, and k = 7 clusters in Proust. Proust clustering results of seven Visium SPG human inferior temporal cortex samples, using five protein channels (DAPI, Abeta, pTau, MAP2, and GFAP), top 30 PCs from reconstructed gene expression, top 5 PCs from reconstructed extracted image features, and $k = 7$ clusters in Proust.

bioRxiv preprint doi: https://doi.org/10.1101/2024.02.02.578662; this version posted February 4, 2024. The copyright holder for this preprint (which was not certified by peer review) is the author/funder, who has granted bioRxiv a license to display the preprint in perpetuity. It is made available under aCC-BY 4.0 International license.

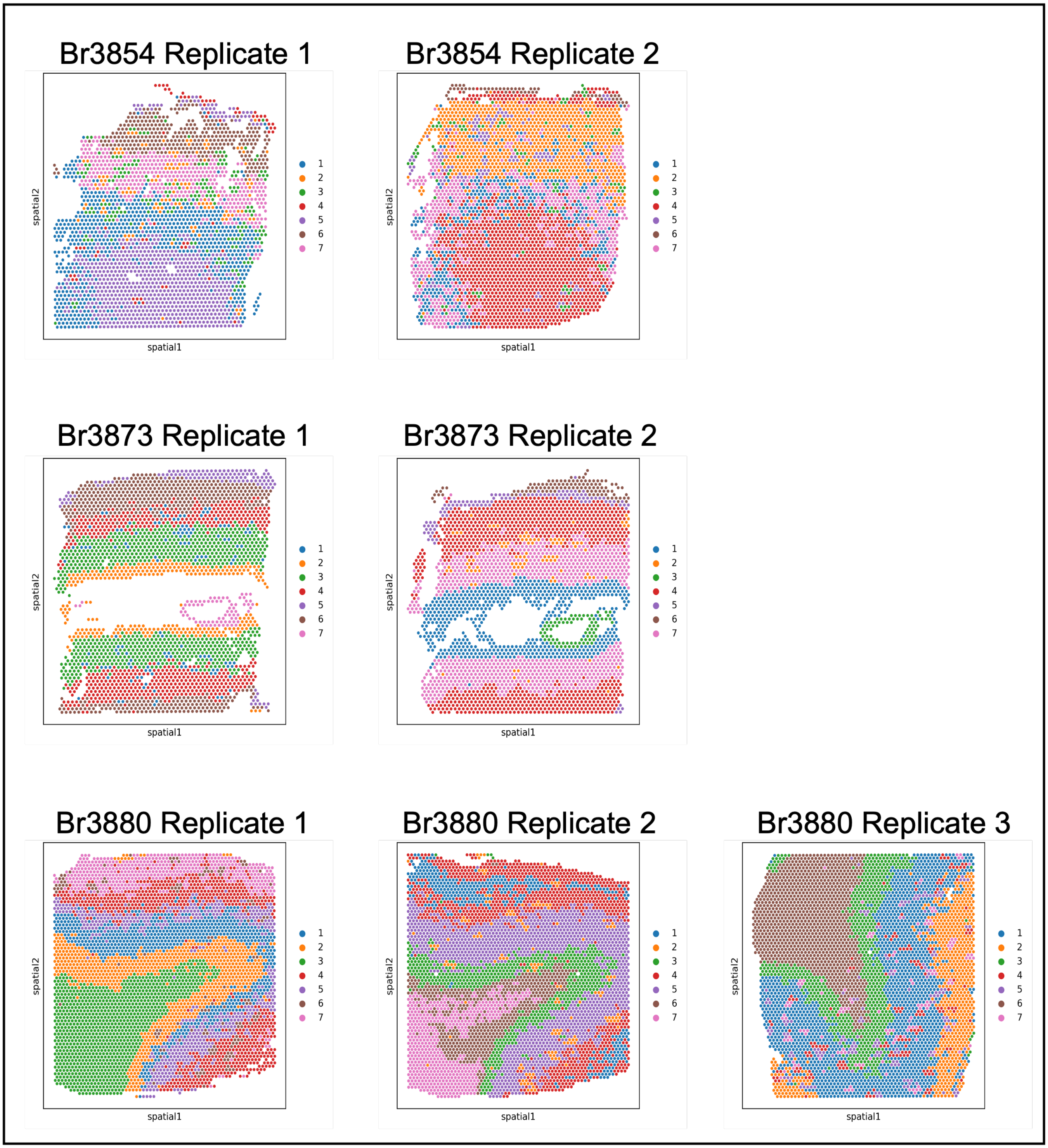

Figure S9: Proust clustering results of the Visium SPG human inferior temporal cortex samples, using two protein channels (Abeta and pTau), top 10 PCs from reconstructed gene expression, top 10 PCs from reconstructed extracted image features, and k = 7 clusters in Proust. Proust clustering results of seven Visium SPG human inferior temporal cortex samples, using two protein channels (Abeta and pTau), top 10 PCs from reconstructed gene expression, top 10 PCs from reconstructed extracted image features, and $k = 7$ clusters in Proust.

bioRxiv preprint doi: https://doi.org/10.1101/2024.02.02.578662; this version posted February 4, 2024. The copyright holder for this preprint (which was not certified by peer review) is the author/funder, who has granted bioRxiv a license to display the preprint in perpetuity. It is made available under aCC-BY 4.0 International license.

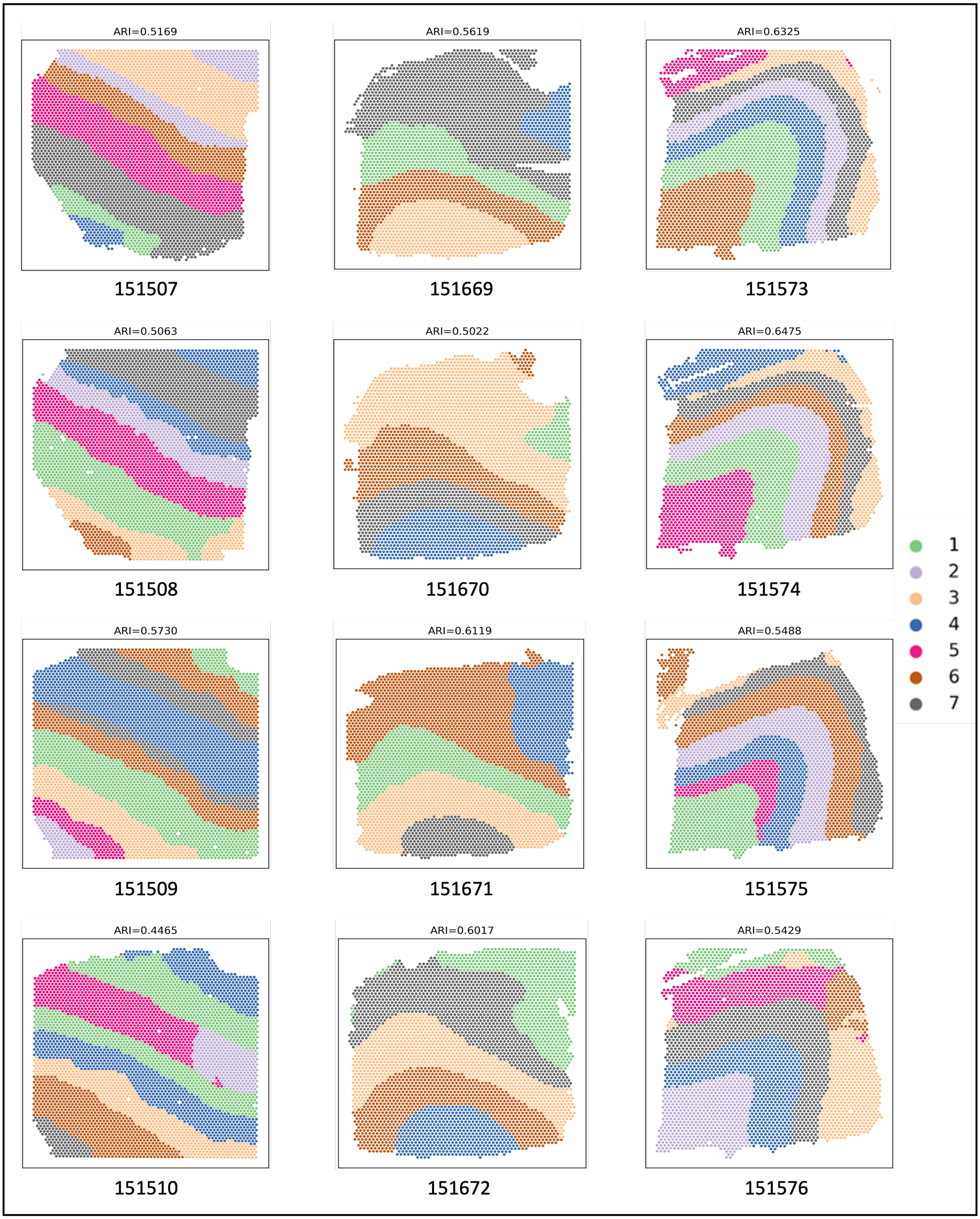

Figure S10: Proust clustering results of the Visium human DLPFC samples with H&E images. Proust clustering results of 12 Visium human DLPFC samples that contain H&E images.
